# Identification of biomarkers associated with mitochondrial dysfunction and programmed cell death in chronic obstructive pulmonary disease via transcriptomics

**DOI:** 10.3389/fgene.2025.1567173

**Published:** 2025-06-19

**Authors:** Xiaojuan Yang, Yutao Duan, Lei Qiu, Xia Huang, Fei Li

**Affiliations:** ^1^ Department of Pulmonary and Critical Care Medicine, Shanxi Provincial People’s Hospital, Taiyuan, China; ^2^ Radiology Department, The First People’s Hospital of Jinzhong, Jinzhong, Shanxi, China; ^3^ The Fifth Clinical Medical College of Shanxi Medical University, Taiyuan, China

**Keywords:** chronic obstructive pulmonary disease, mitochondria, programmed cell death, biomarkers, immune infiltration

## Abstract

**Background:**

Research has demonstrated that the homeostasis of mitochondria and programmed cell death (PCD) are intimately linked to chronic obstructive pulmonary disease (COPD). Consequently, identifying biomarkers of COPD from mitochondria-related genes (MRGs) and programmed cell death-related genes (PCD-RGs) is of paramount importance.

**Methods:**

Differentially expressed genes (DEGs) from the GSE42057 dataset and COPD-related genes (COPD-RGs) via weighted gene co-expression network analysis (WGCNA) were intersected with MRGs and PCD-RGs to select candidates. Machine learning identified biomarkers, validated across GSE42057 and GSE94916 datasets. Pathway enrichment, immune infiltration, and drug prediction analyses were performed.

**Results:**

Eight candidate genes were derived from intersecting DEGs, COPD-RGs, MRGs, and PCD-RGs. Five biomarkers (BCL2, CCR7, FAM162A, FOXO1, RPS3) were identified, showing consistent dysregulation in COPD. These biomarkers activated the “ribosome” pathway. CCR7 and FOXO1 correlated positively with naïve B cells, while BCL2 negatively correlated with M0 macrophages. BCL2 exhibited strong binding to dolastatin 10, beauvericin, and micellar paclitaxel. RT-qPCR confirmed biomarker expression.

**Conclusion:**

BCL2, CCR7, FAM162A, FOXO1, and RPS3 are biomarkers for COPD, providing a new breakthrough point for the treatment of this disease.

## 1 Introduction

Chronic Obstructive Pulmonary Disease (COPD) is a chronic and frequently progressive lung disorder that exhibits high morbidity and mortality rates (Lareau et al., 2019). It is characterized by persistent airflow limitation and respiratory symptoms, including dyspnea, cough, and sputum production. Despite being preventable and treatable, COPD poses a significant health, economic, and social burden. According to the 2018 China Pulmonary Health Study, there are nearly 100 million COPD patients in China. Despite the ongoing introduction of combination therapy involving inhaled corticosteroids for COPD treatment, the control rate and mortality of the disease continue to increase significantly, and its pathogenesis remains incompletely understood (Xu et al., 2023; Baltazar-Garcia et al., 2024; De Miguel-Diez et al., 2024).

Mitochondria are well-established organelles that maintain cellular bioenergetics by producing ATP. Although oxidative phosphorylation may be their most important function, mitochondria are also integral for the synthesis of metabolic precursors, calcium regulation, the production of reactive oxygen species, immune signaling, and apoptosis. Mitochondria-dependent pathways may represent a promising therapeutic target for alleviating human diseases (Harrington et al., 2023). Over the past decade, accumulating experimental evidence has demonstrated that mitochondrial dysfunction plays a pivotal role in the pathogenesis of chronic lung diseases, such as COPD (Ryter et al., 2018; Roque et al., 2020; Mora et al., 2017). However, the specific mechanisms and detectable molecular targets of mitochondrial dysfunction in COPD remain unclear. High-throughput sequencing data can be utilized to construct and validate clinical prediction models for COPD using bioinformatics methods, offering a new reference for its clinical diagnosis and treatment (Pokharel et al., 2024; Liu et al., 2024).

Programmed cell death (PCD) occurs as part of normal physiologic processes, including organ development and epithelial renewal. It also occurs in cells that cannot mitigate stressors threatening tissue homeostasis (Bedoui et al., 2020; Tower, 2015). Dysregulated PCD has been implicated in human diseases (Sauler et al., 2019; Mizumura et al., 2014). Excessive PCD results in tissue injury and destruction, while the dysregulateion of PCD is associated with mutagenesis, impaired immunity, and autoimmune disease (Camilli et al., 2021). The regulation of cell death is particularly critical in the lung, an organ required to maintain a delicate network of epithelial-endothelial interfaces for effective gas exchange of CO2 for O2, all while being exposed to environmental stressors such as pathogens and aerosolized toxins. Multiple studies have demonstrated increased apoptosis in the lungs of patients with COPD (Sauler et al., 2019). Numerous studies have established that mitochondria play a pivotal role in the process of apoptotic cell death. Numerous studies have demonstrated that mitochondria play a pivotal role in apoptotic cell death. This study aims to contribute to the clinical diagnosis and treatment of COPD by identifying biomarkers associated with mitochondrial dysfunction and programmed cell death in patients with COPD.

In this study, we employed transcriptome data analysis, Weighted Gene Co-expression Network Analysis (WGCNA), machine learning, and other methodologies. A nomogram model, based on these biomarkers, was constructed and validated to serve as a predictive tool for COPD incidence. The exploration of biomarker-based Gene Set Enrichment Analysis (GSEA) and GeneMANIA, along with the construction of regulatory networks such as competing endogenous RNA (ceRNA), holds significant potential for further elucidating the pathogenesis of COPD.

## 2 Materials and methods

### 2.1 Data source

The GSE42057 (training dataset) and GSE94916 (validation dataset) datasets were retrieved from the Gene Expression Ontology database (GEO, http://www.ncbi.nlm.nih.gov/geo/) (Song et al., 2024). The GSE42057 dataset (GPL570) contained 94 peripheral blood samples of chronic obstructive pulmonary disease (COPD) patients and 42 peripheral blood samples from healthy controls (controls) (Bahr et al., 2013; Bowler et al., 2015; Cruickshank-Quinn et al., 2018; Bowler et al., 2013). Peripheral blood samples from six COPD patients and six healthy controls (controls) were screened in the GSE94916 dataset (GPL20844). In all 2,030 mitochondrial-related genes (MRGs) obtained from the MitoCarta 3.0 database (https://www.broadinstitute.org/mito) (Zhang L. et al., 2024). A total of 1,548 programmed cell death-related genes (PCD-RGs) were retrieved from the literature (Qin et al., 2023).

### 2.2 Selection of candidate genes

Based on the GSE42057 dataset, differentially expressed genes (DEGs) in COPD and control were screened *via* the ‘limma’ (version 3.46.0) (Ritchie et al., 2015) under the screening criteria of p < 0.05 (Liu et al., 2022). At the same time, the GSE42057 dataset was analyzed for weighted gene co-expression network analysis (WGCNA) (Zhang and Horvath, 2005) with the aim of obtaining key modules related to COPD. The steps to do this were to remove outlier samples in the GSE42057 dataset through cluster analysis. Next, the soft threshold (β) was determined by setting the scale-free *R* ^2^ to be greater than 0.85 and the average connectivity tended to be 0. Then, similarly expressed genes were incorporated into the same gene modules by a dynamic tree-cutting method (minModuleSize = 30, mergeCutHeight = 0.4). Subsequently, the modules most significantly associated with COPD were screened as key modules based on the correlation coefficients (|cor| > 0.3, p < 0.05), and the genes in them were recorded as COPD-related genes (COPD-RGs). Ultimately, the intersection of DEGs, COPD-RGs, 2,030 MRGs, and 1,548 PCD-RGs was obtained and partially recorded as candidate genes for COPD.

### 2.3 Biological function analysis of candidate genes and construction of their protein-protein interaction (PPI) networks

The candidate genes were subjected to Gene ontology (GO) and Kyoto Encyclopedia of Genes and Genomes (KEGG) enrichment analysis (*p* < 0.05) using the ‘clusterProfiler’ (version 4.4.4) (Yu et al., 2012). Meanwhile, the interaction information of the candidate genes at the protein level was obtained with the help of the search tool for recurring instances of neighbouring genes (STRING, https://string-db.org/) (Liu et al., 2021), and the PPI network was constructed (low confidene = 0.15) (Garcia-Jimenez et al., 2010).

### 2.4 Identification of biomarkers for COPD

In order to obtain the characteristic genes of COPD, the candidate genes were subjected to least absolute shrinkage and selection operator (LASSO) analysis by the ‘glmnet’ (version 4.1–6) (Yang L. et al., 2022) based on the optimal coefficients and lambda values. Meanwhile, Support Vector Machine Recursive Feature Elimination (SVM-RFE) was used to determine the best screening variables by eliminating the feature vectors generated by SVM (Sanz et al., 2018). In addition, the Boruta algorithm was used to calculate the characteristics of the candidate genes, and the most important characteristic genes were screened by sorting them according to their characteristics (Zhou et al., 2023). Based on the above three machine learning algorithms, the intersecting genes obtained from three machine learning algorithms were deemed as the biomarkers for COPD. Finally, based on the GSE42057 and GSE94916 datasets, the expression levels of the biomarkers were detected and validated between COPD and controls.

### 2.5 Construction of a nomogram model for COPD

In order to evaluate the use of biomarkers for predicting the probability of COPD, it was necessary to construct a nomogram model based on biomarkers in the GSE42057 dataset. Furthermore, calibration curves (Austin et al., 2020), decision curve analysis (DCA) (Vickers and Holland, 2021), and clinical impact curve (CIC) were used to demonstrate the validity of the nomogram for the prediction of COPD patients.

### 2.6 Gene set enrichment analysis (GSEA) of biomarkers

In the GSE42057 dataset, correlation coefficient between biomarkers and other gene were calculated and ranked in descending order. The biomarkers were then subjected to GSEA (Duan et al., 2023) using the ‘clusterProfiler’. The reference gene set was the background gene set ‘c2. cp.kegg_legacy.v2023.2. Hs.symbols.gmt’ from the Molecular Signatures Database (MSigDB) (Guo et al., 2023) (p < 0.05).

### 2.7 Construction of gene interaction network and regulatory network

GeneMANIA (http://genemania.org/) was used to explore interactions between biomarkers and other functionally similar genes and to construct co-expression networks between them. In order to investigate the regulatory mechanisms of biomarkers at the molecular level, the miRnet database (http://mirdb.org) (Wong and Wang, 2015) and ‘multiMiR’ (org = ‘hsa’, table = ‘validated’) (version 1.16.0) (Hosseinpour et al., 2023) were used to predict the upstream miRNAs corresponding to biomarkers, respectively. And miRNAs were then obtained by taking the intersection of the predictions from the above two results. After that, the upstream lncRNAs (clipExpNum >5) of the miRNAs were predicted using the Starbase database (https://starbase.sysu.edu.cn/) (Liu J. et al., 2023). Moreover, circRNAs targeting miRNAs were also predicted using the circbank database (http://www.circbank.cn/) (Su et al., 2023). Based on this, a lncRNA-miRNA-mRNA-cirRNA regulatory network was constructed using the ‘Cytoscape’ (version 3.8.2) (Shannon et al., 2003).

### 2.8 Immune infiltration analysis

The relative proportions of 22 immune cells per sample in the COPD and controls of the GSE42057 dataset were calculated by the Cell-type Identification By Estimating Relative Subsets Of RNA Transcripts (CIBERSORT) algorithm (Tao et al., 2023). Then, the differences between COPD and controls of these immune cells were compared by Wilcoxon’s test (*p* < 0.05). Next, Spearman’s correlation analysis was used to examine the relationship between significantly differentially immune cells and biomarkers (|cor| > 0.3, *p* < 0.05) (Yang F. et al., 2022).

### 2.9 Association of biomarkers with COPD and potential drug prediction

Furthermore, inference score between biomarkers and COPD were evaluated in the comparative toxicogenomics database (CTD, http://ctdbase.org) (Davis et al., 2023) with the aim of exploring the correlation between biomarkers and COPD. Meanwhile, the Drug-Gene Interaction database (DGIdb, http://dgidb.org/) (Zhang Y. et al., 2024) online database was used to predict the targeted drugs associated with the biomarkers, and the targeting relationships between the biomarkers and drugs were demonstrated using ‘Cytoscape’. More investigation into their molecular docking was required to comprehend the molecular interactions between drugs and biomarkers. First, the PDB files of biomarkers were downloaded from Universal Protein (UniProt, https://beta.uniprot.org) (Ostberg et al., 2024). Then, the 2D structure of the drug was converted into a 3D structure using the ‘pybel’ (version 3.0) (O'Boyle et al., 2008). And it was also possible to download the 3D structure of the drug from the PubChem (https://pubchem.ncbi.nlm.nih.gov/) (Huang et al., 2024). Next, they were prepared and pre-processed for drug and biomarker protein structures using ‘AutoDock’ (version 1.5.6) (Zhang et al., 2022). Immediately after that, they were molecularly docked using ‘AutoDock Vina’ (version 1.2.3) (Zhang J. C. et al., 2024). Finally, the results of molecular docking were visualized with the help of ‘Pymol’ (version 2.2.0) (Lv et al., 2024).

### 2.10 Expression analysis of biomarkers

For validating the expression of biomarkers, reverse transcription quantitative polymerase chain reaction (RT-qPCR) was employed on 10 COPD and 10 control PBMC samples in Shanxi Provincial People’s Hospital, China. This study was approved by the Medical Ethics Committee of Shanxi Provincial People’s Hospital (No. 487). All patients had signed an informed consent form. The amplification conditions for RT-qPCR were 40 cycles with 1 min at 95°C, 20 s at 95°C, 20 s at 55°C, and 30 s at 72°C. The qPCR primers were listed in Supplementary Table S1 with GAPDH as reference gene. The relative expression levels of biomarkers were calculated using the 2^-△△CT^ method.

### 2.11 Statistical analysis

The R programming language (version 4.2.2) was used for all analyses, and the Wilcoxon’s test was employed to evaluate the data from various groups. A *p*-value of less than 0.05 was deemed to be statistically significant, unless otherwise noted.

## 3 Results

### 3.1 A total of eight candidate genes were obtained

**FIGURE 1:**
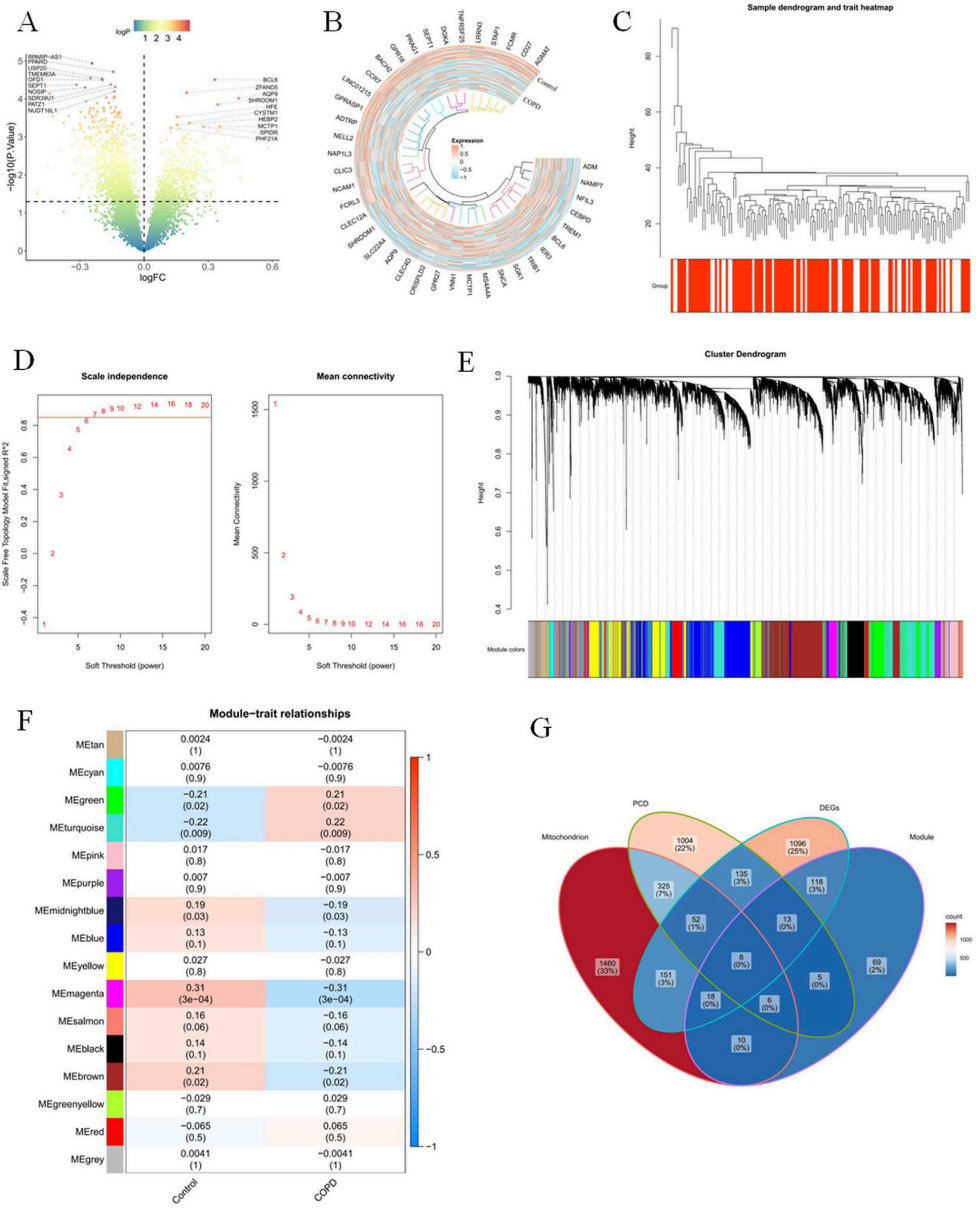
Eight candidate genes were obtained **(A)** In the dataset GSE42057, a volcano plot of the differences in sequencing data of peripheral blood from healthy donors and COPD patients. **(B)** Heat map display of genes with significant differences in the dataset GSE42057. **(C)** Cluster analysis of all samples in the dataset, presenting the outlier sample GSM1031656. In the WGCNA analysis, R2 value screening **(D)** and gene module classification **(E)**. **(F)** Heat map of the gene module. **(G)** Differential analysis and the intersection Venn diagram of COPD-related gene modules.

The expression of 480 DEGs was upregulated, and 111 DEGs were downregulated out of 1,591 DEGs obtained in the GSE42057 dataset. The distribution of these DEGs was visualised by volcano and heatmap (Figures 1A, B). Cluster analysis of all samples in the GSE42057 dataset showed that the GSE42057 dataset could continue to be used for subsequent analyses after removing the outlier sample GSM1031656 (Figure 1C). When *R* ^2^ reaches 0.85 and the average connectivity tends to zero, β was 7 (Figure 1D). Then, a total of 16 co-expressed gene modules with different colors were screened (Figure 1E). Among them, the MEmagenta module with the most significant correlation with the COPD was served as a key module (cor = 0.31, *p* < 0.05), and it contained 247 COPD-RGs (Figure 1F). Based on the above results, the intersections of 1,591 DEGs, 247 COPD-RGs, 2,030 MRGs, and 1,548 PCD-RGs were taken to obtain a total of eight candidate genes (BAG3, BCL2, CCR7, FAM162A, FOXO1, RPS3, SLC39A8, and HINT1) (Figure 1G).

### 3.2 Exploration of potential biological functions and signalling pathways of candidate genes

**FIGURE 2:**
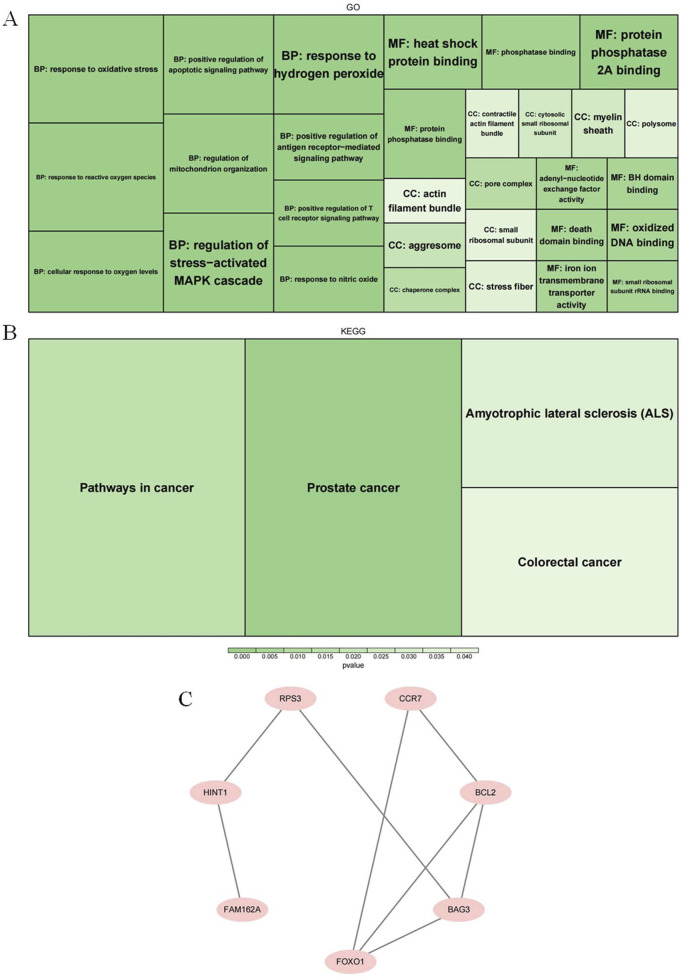
Exploration of potential biological functions and signaling pathways of candidate genes **(A)** A display diagram of the significance pathways in GO enrichment analysis. **(B)** A display diagram of the significance pathways of KEGG enrichment analysis. **(C)** The protein-protein interaction map of the genes we screened.

GO results showed that the candidate genes were annotated for 552 biological processes (BPs), 15 cellular components (CCs), and 45 molecular functions (MFs). The BPs in which candidate genes significantly enriched contained ‘response to oxidative stress”, “response to reactive oxygen species”,” regulation of stress-activated. MAPK cascade’, and ‘regulation of mitochondrion organization’ and so on (*p* = 0.005). The CC items which were involved by candidate genes comprising ‘pore complex’, ‘chaperone complex’, and ‘aggresome’ and so on (*p* < 0.02). In regard to MFs, candidate genes were mainly involved in the ‘heat shock protein binding’, ‘protein phosphatase binding’, ‘protein phosphatase 2 A binding’, and ‘iron ion transmembrane transporter activity’ and so on (*p* = 0.005) (Figure 2A). The signaling pathways where KEGG results showed significant enrichment of candidate genes were ‘prostate cancer’, ‘pathways in cancer’, ‘amyotrophic lateral sclerosis (ALS)’, and ‘colorectal cancer’ (*p* < 0.05) (Figure 2B). Protein interactions were demonstrated in the PPI network with eight protein nodes and eight edges. Among them, the protein level interactions between FOXO1 and BAG3, BCL2 and CCR7 were closer (Figure 2C).

### 3.3 BCL2, CCR7, FAM162A, FOXO1, and RPS3 identified as biomarkers of COPD

**FIGURE 3:**
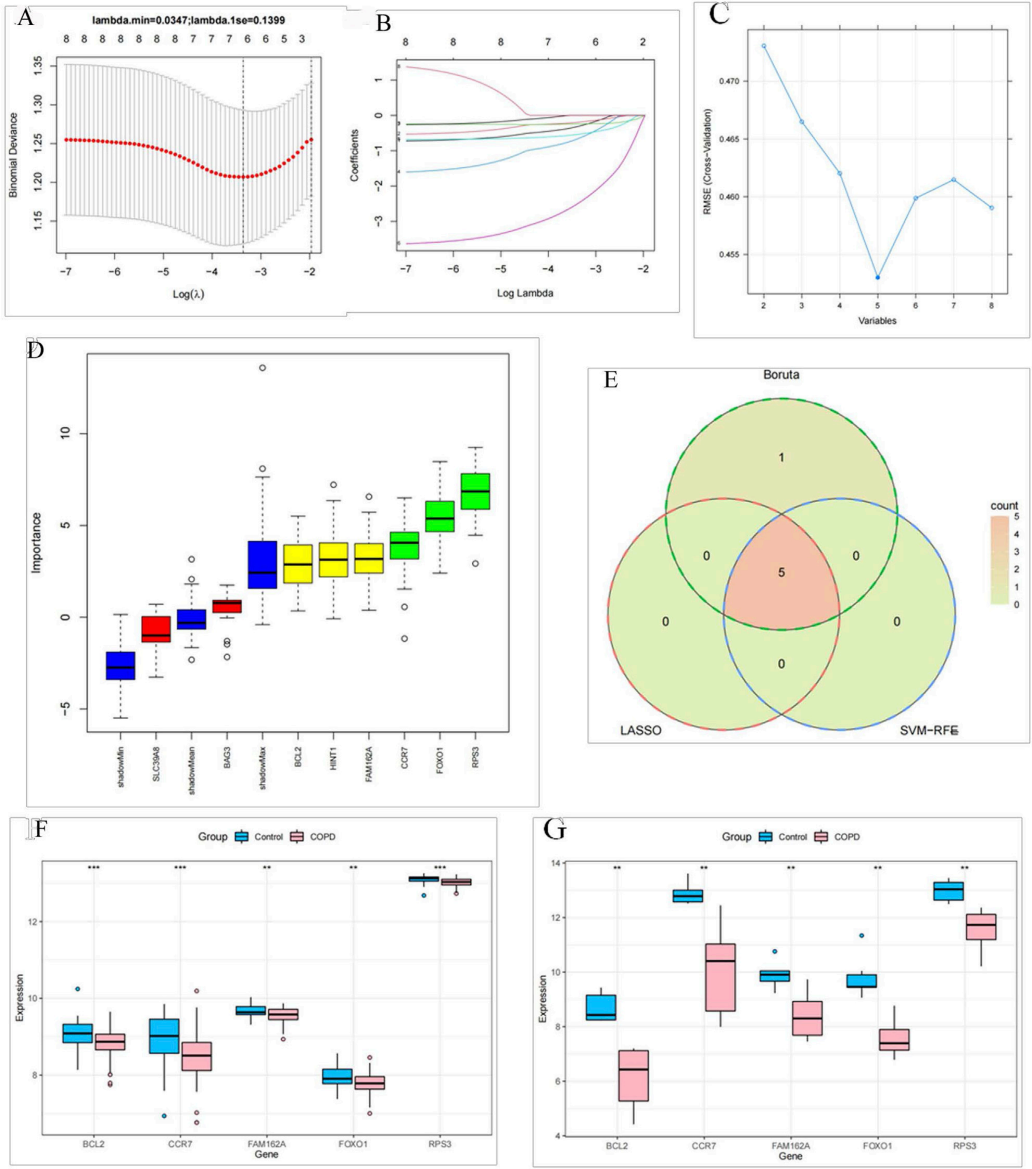
BCL2, CCR7, FAM162A, FOXO1, and RPS3 identified as biomarkers of COPD **(A)** Cross-validation curve of LASSO regression. The X-axis represents the logarithm of the penalty coefficient, log Λ, and the Y-axis represents the likelihood deviation. The smaller the Y-axis, the better the fitting effect of the equation. **(B)** LASSO regression coefficient path diagram. We included seven variables. That is, each curve represents the change trajectory of each independent variable coefficient. The vertical coordinate is the value of the coefficient, and the lower horizontal coordinate is log(λ). **(C)** The error rate curve based on the 5-fold cross-validation of the SVM-RFE algorithm. **(D)** A box plot in which the Z-Scores calculated by the machine learning Feature Selection algorithm Boruta are sorted in the order of the original variables. **(E)** The intersection Venn diagram of three analytical methods. The five genes of the intersection results were differentially expressed in healthy individuals and COPD patients in the GSE94916 dataset **(F)** and the GSE42057 dataset **(G)**.

BAG3, BCL2, CCR7, FAM162A, FOXO1, and RPS3 were selected as characteristic genes when the minimum value of lambda was 0.03 in the LASSO analysis (Figures 3A, B). Meanwhile, the SVM-RFE algorithm identified the five most important characteristic genes (RPS3, FOXO1, CCR7, FAM162A, and BCL2) (Figure 3C). The Boruta algorithm filtered out six characteristic genes (BCL2, CCR7, FAM162A, FOXO1, RPS3, and HINT1) (Figure 3D). Immediately after, the intersection of results from LASSO, SVM-RFE and Boruta algorithms yielded BCL2, CCR7, FAM162A, FOXO1, and RPS3 as biomarkers for COPD (Figure 3E). Additionally, these five biomarkers were significantly downregulated in COPD based on the GSE94916 dataset. And their expression trends were consistent with the GSE42057 dataset (Figures 3F,G).

### 3.4 The nomogram model was capable of predicting the incidence of COPD

**FIGURE 4:**
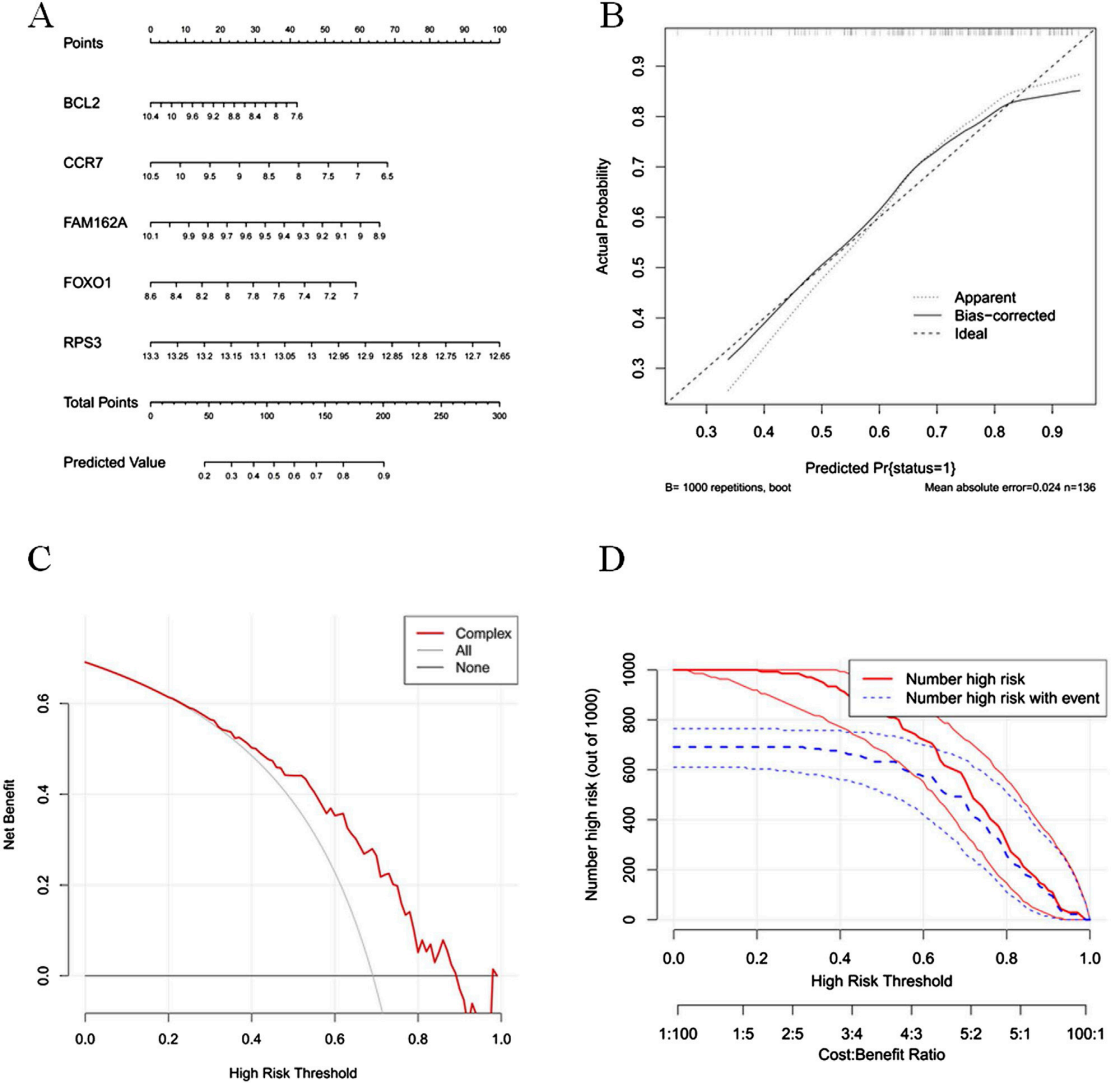
The nomogram model was capable of predicted the incidence of COPD **(A)** The nomogram model was constructed based on BCL2, CCR7, FAM162A, FOX01 and RPS3. **(B)** The calibration curves for the nomogram. The x-axis represents the nomogram-predicted probability and y-axis represents the actual probability of invasive adenocarcinoma. **(C)** Decision curve analysis (DCA) evaluating the clinical utility of predictive models. The decision curve illustrates the net benefit across a range of threshold probabilities for model. The x-axis represents the threshold probability—the risk level at which a patient would opt for intervention. The y-axis shows the net benefit, calculated by weighting the true positives against false positives. **(D)** Clinical impact curve (CIC) of the predictive model. The clinical impact curve shows the number of individuals classified as high risk by the prediction model at each threshold probability (red curve), and among them, the number of true positives (blue curve).

The nomogram model was constructed based on BCL2, CCR7, FAM162A, FOXO1, and RPS3, which was effective in predicting the incidence of COPD patients (Figure 4A). The results of the calibration curve showed that the predicted with actual probabilities of the model was closed to Y = X, which indicated a good calibration of the model (Figure 4B). And the DCA results showed that the net benefit of the model was higher than that of the biomarkers, which further proved that the model has a good prospect for clinical application (Figure 4C). The clinical validity of the model was further demonstrated by the CIC results which showed that the number of high-risk individuals and the number of true positives were consistent when the threshold probability exceeds 60% (Figure 4D).

### 3.5 Functional annotation of five biomarkers

**FIGURE 5:**
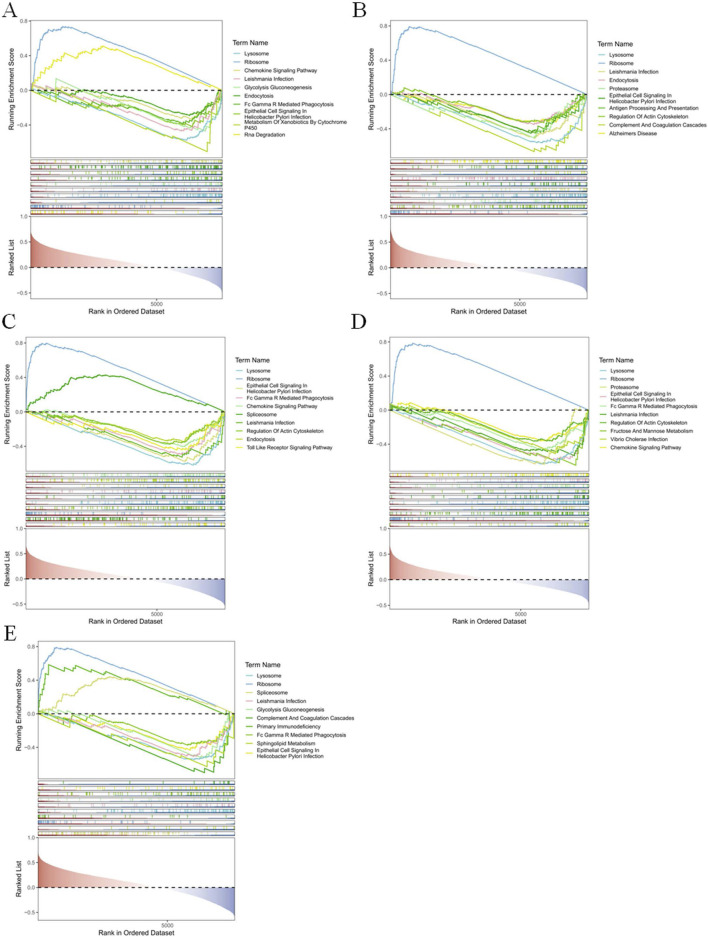
Function of BCL2, CCR7, FAM162A, FOXO1, and RPS3 In the dataset GSE42057, we analyzed the top ten pathway maps based on the differences of the screened genes BCL2 **(A)**, CCR7 **(B)**, FAM162A **(C)**, FOXO1 **(D)**, and RPS3 **(E)** through GSEA analysis.

GSEA results showed that the signaling pathways co-enriched by these five biomarkers were ‘Ribosome’, ‘Lysosome’, ‘Leishmania Infection’, ‘Endocytosis’, and ‘Epithelial Cell Signaling In’ and so on. Among them, BCL2 enriched ‘Ribosome’ and ‘Rna Degradation’ were significantly upregulated and other signaling pathways were significantly downregulated (Figure 5A). Only ‘Ribosome’ of the signaling pathways enriched in CCR7 was significantly upregulated (Figure 5B). Similarly, Ribosome’ was significantly upregulated in FAM162A, FOXO1, and RPS3 (Figures 5C–E).

### 3.6 Functional associations of biomarkers and their potential molecular regulation

**FIGURE 6:**
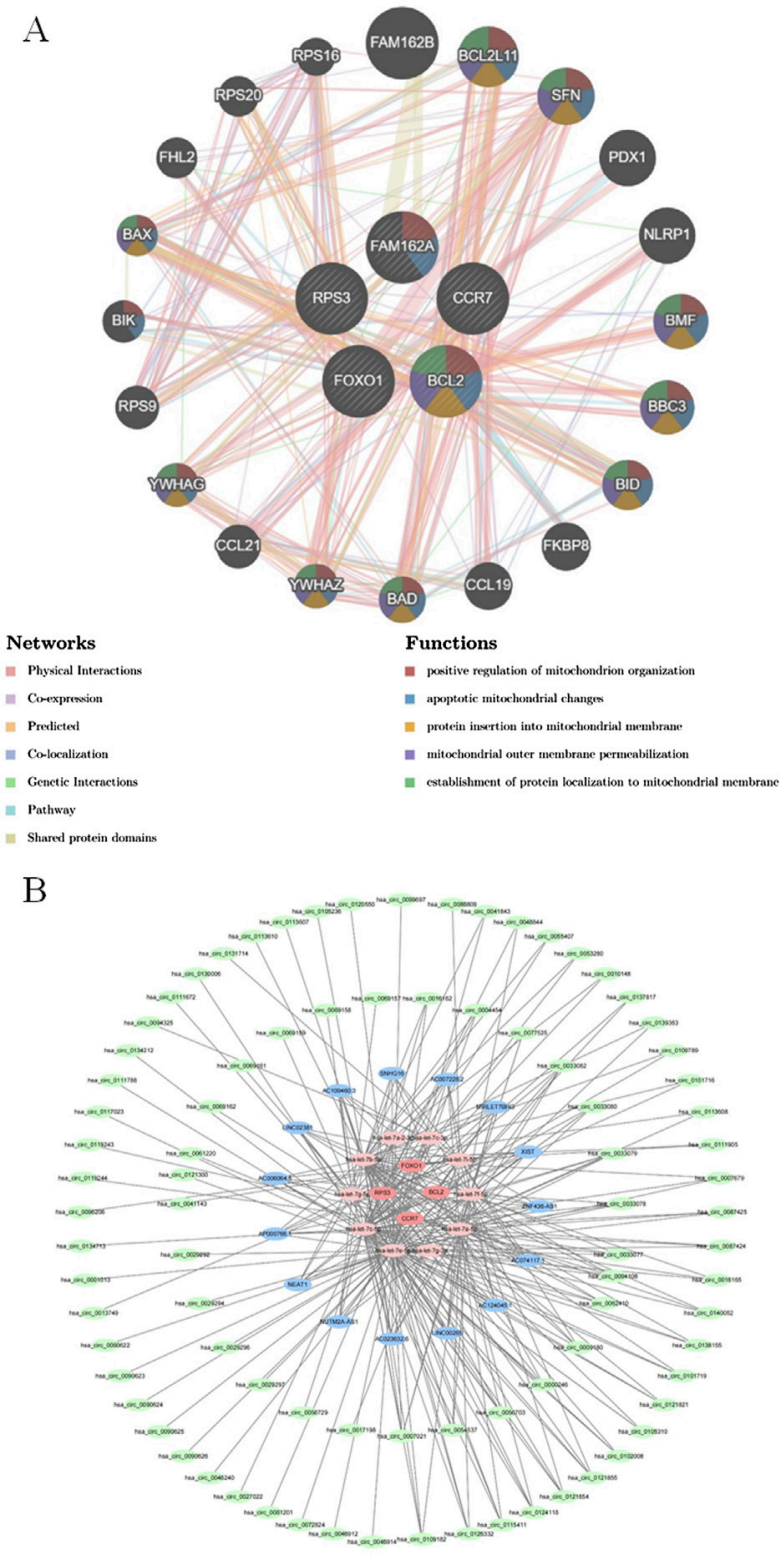
Functional associations of biomarkers and their potential molecular regulation **(A)** Gene interaction networks of the five candidate genes generated by GeneMANIA. The interaction network illustrates the functional associations among the five input genes and their related genes, just as predicted by GeneMANIA. Nodes represent genes, and the colored lines (edges) indicate different types of predicted interactions, including co-expression, physical interactions, co-localization, and shared protein domains. **(B)** Construction of the ceRNA network based on miRNet and multiMiR prediction results. The ceRNA (competing endogenous RNA) network was constructed using five candidate mRNAs and their associated miRNAs predicted by miRNet and multiMiR databases.

The GeneMANIA results showed that there were various relationships between biomarkers and other genes such as co-expression, the same location, physical interactions, genetic interactions, sharing protein structural domains, or participating in the same pathway. Among the biological functions shared by these five biomarkers were ‘positive regulation of mitochondrion organization’, ‘establishment of protein localization to mitochondrial membrane’, and ‘protein insertion into mitochondrial membrane’. The same biological functions and roles were also observed between BCL2 and BCL2L11, SFN, BMF, BBC3, BID, BAD, YWHAZ, YWHAG, and BAX, respectively (Figure 6A). Furthermore, the results from miRnet and multiMiR prediction were intersected and a total of 10 miRNAs were obtained. Subsequently, a total of 151 target lncRNAs and 180 target circRNAs were predicted based on the 10 miRNAs, respectively. Based on this, a ceRNA regulatory network for COPD was constructed (Figure 6B). Among them, the miRNAs shared by these five biomarkers were hsa-let-7a-2-3p, hsa-let-7c-3p, hsa-let-7i-5p, hsa-let-7f-5p, hsa-let-7a-5p, hsa-let-7g-3p, hsa-let-7e-5p, hsa-let-7c-5p, hsa-let-7g-5p, and hsa-let-7b-5p. Of these, hsa-let-7e-5p predicted the most lncRNAs and RNAs.

### 3.7 M0 macrophages, neutrophils and naive B cells were significantly different in COPD and controls

**FIGURE 7:**
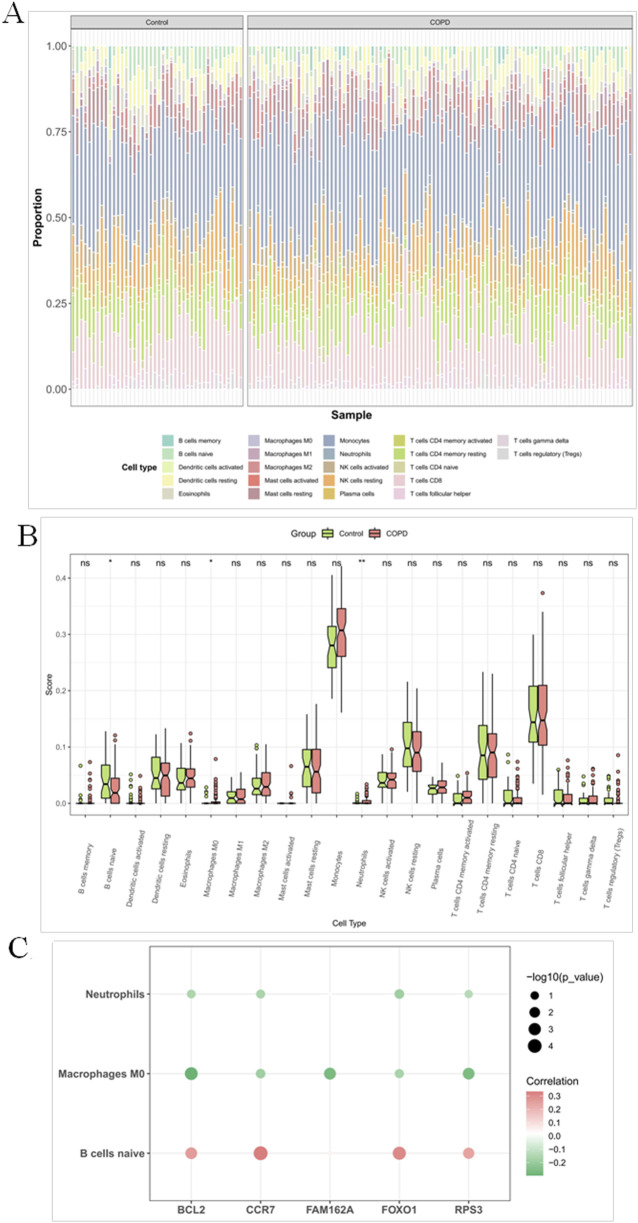
M0 macrophages, neutrophils and naive B cells were significantly different in COPD and controls **(A)** Heatmap of immune cell proportions in peripheral blood samples from COPD patients and healthy controls estimated by CIBERSORT. The heatmap displays the relative proportions of 22 immune cell types inferred by the CIBERSORT algorithm in each sample. Columns represent individual peripheral blood samples from the COPD group and normal control group, while rows indicate distinct immune cell subtypes. **(B)** Bar plot of differential immune cell proportions between COPD patients and healthy controls based on CIBERSORT analysis. Each bar represents the mean proportion of a specific immune cell subtype in each group, with error bars indicating standard error of the mean (SEM). Asterisks denote statistically significant differences between groups (*P < 0.05, **P < 0.01, ***P < 0.001), determined by the Wilcoxon rank-sum test. **(C)** Correlation heatmap between five candidate genes and immune cell proportions estimated by CIBERSORT. The heatmap illustrates the correlations between the expression levels of five selected genes and the relative abundances of immune cell types inferred by the CIBERSORT algorithm. Each dot represents a gene–cell type pair. The color and numeric value indicate the strength and direction of the correlation (Spearman’s r), while the dot size reflects statistical significance, represented as -log_10_(P value). Larger dots denote more statistically significant associations.

The proportion of immune cell distribution was different for individual samples in the GSE42057 dataset (Figure 7A). Among them, M0 macrophages and neutrophils were significantly upregulated in COPD, while naive B cells were significantly downregulated in COPD (Figure 7B). Further correlation results showed that CCR7 and FOXO1 were significantly positively correlated with naive B cells, whereas BCL2 was significantly negatively correlated with M0 macrophages (Figure 7C).

### 3.8 Standard binding capacity between the biomarkers and drugs

**FIGURE 8:**
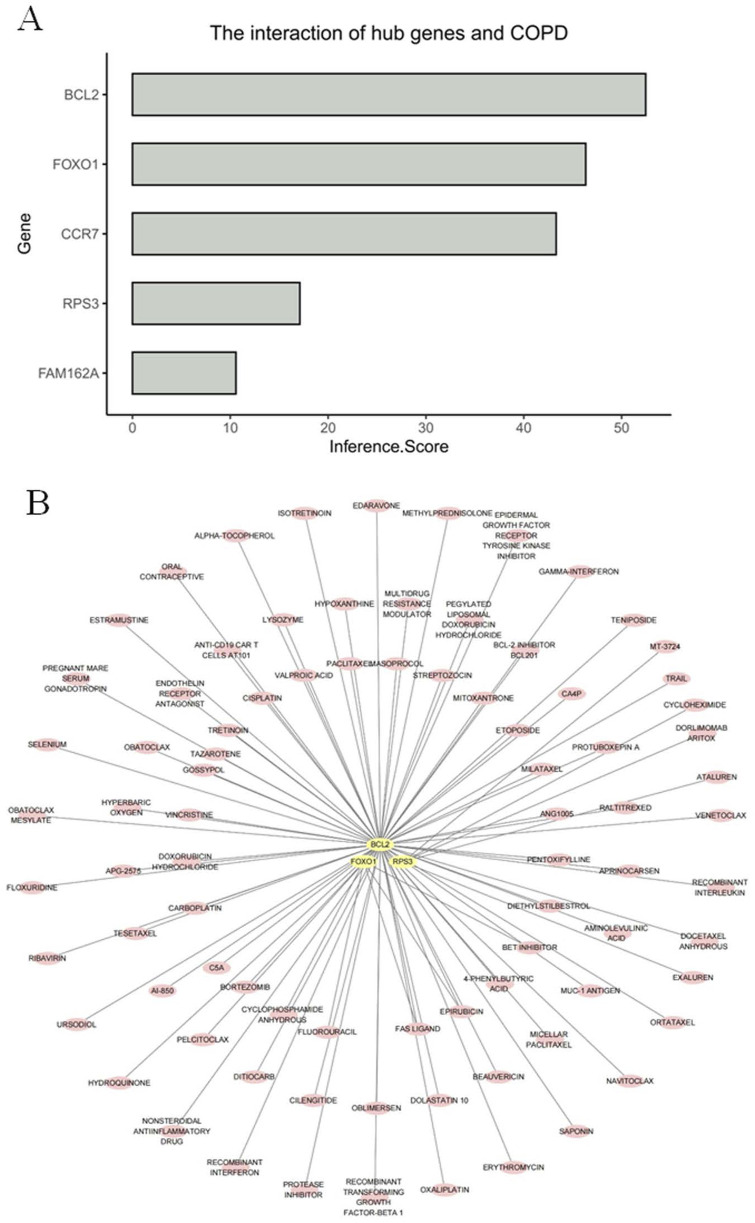
Standard binding capacity between the biomarkers and drugs **(A)** Bar plot of inferred association scores between five candidate genes and COPD. The bar chart illustrates the inference scores of five candidate genes in relation to COPD. The inference score on the x-axis reflects the strength of the predicted association, as derived from integrated databases or computational inference models. The y-axis lists the five candidate genes. **(B)** Drug–gene interaction network for three candidate genes based on the DGIdb database. The network illustrates predicted or known interactions between three candidate genes and their corresponding drugs, as identified through the Drug–Gene Interaction Database (DGIdb).

Among the results of the inference scores between biomarkers and COPD, BCL2, CCR7, and FOXO1 showed the strongest association with COPD (Figure 8A). In addition, the drug prediction results showed that the biomarkers predicted a total of 86 drugs for COPD treatment (Figure 8B). Among them, RPS3 had the strongest binding to ataluren (affinity = −6.5), and cycloheximide (affinity = −6.4), respectively. FOXO1 only binds most strongly to epirubicin (affinity = −7.5). In contrast, BCL2 showed strongest bonding capability to dolastatin 10 (affinity = −11.5), beauvericin (affinity = −8.5), and micellar paclitaxel (affinity = −11.5) (Table 1). In addition, the 3D map between biomarkers and drugs visualized the binding sites between them (Supplementary Figure S1).

**TABLE 1:** Molecular docking results

Gene	Drug	COMPOUND CID	Molecular docking binding energy (Kcal/mol)
RPS3(PDBID:1WH9)	EXALUREN	71461382	-4.22
RPS3(PDBID:1WH9)	ATALUREN	11219835	-6.5
RPS3(PDBID:1WH9)	CYCLOHEXIMIDE	6197	-6.4
FOXO1(PDBID:8A65)	EPIRUBICIN	41867	-7.5
FOXO1(PDBID:8A65)	CYCLOPHOSPHAMIDE ANHYDROUS	2907	-4.1
FOXO1(PDBID:8A65)	FLUOROURACIL	3385	-4.2
BCL2(PDBID:2W3L)	OBLIMERSEN	118984457	15.71
BCL2(PDBID:2W3L)	DOLASTATIN 10	9810929	-11.5
BCL2(PDBID:2W3L)	BEAUVERICIN	3007984	-8.5
BCL2(PDBID:2W3L)	MICELLAR PACLITAXEL	49881022	-11.5

### 3.9 All biomarkers were significantly downregulated in COPD

**FIGURE 9:**
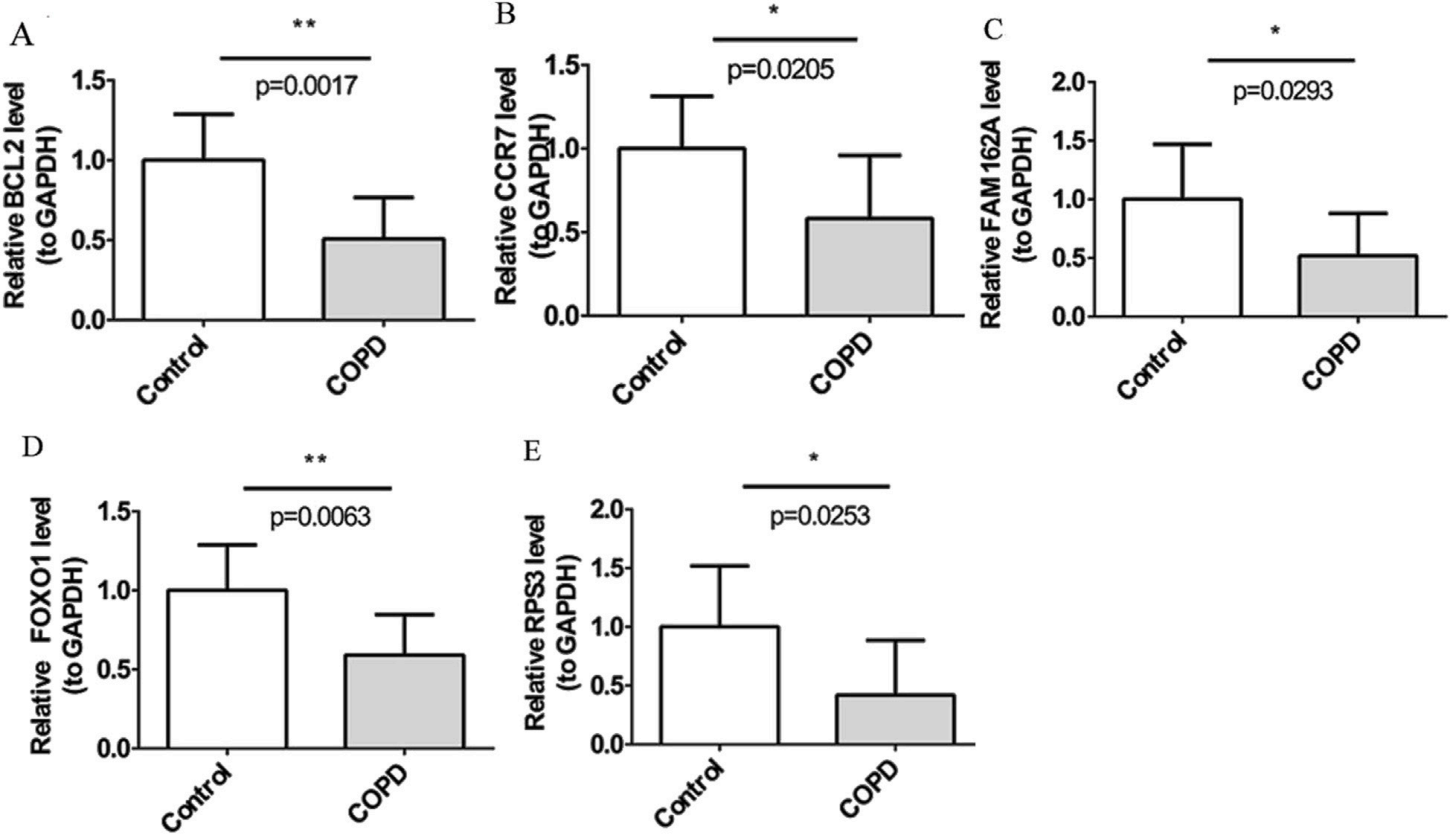
BCL2, CCR7, FAM162A, FOXO1, and RPS3, were down-regulated in COPD by RT-PCR Quantitative PCR analysis of five candidate genes in peripheral blood samples of patients with chronic obstructive pulmonary disease and healthy controls, including BCL2 **(A)**, CCR7 **(B)**, FAM162A **(C)**, FOXO1 **(D)**, and RPS3 (E). The bar plot displays the relative expression levels of five candidate genes, normalized to GAPDH, in the control group and the COPD group. The y-axis represents relative gene expression levels (compared to GAPDH), and the x-axis shows the two groups. Each bar represents the mean ± standard error of the mean (SEM) for each gene. Statistical significance between groups was assessed using an unpaired two-tailed t-test. P values are indicated as follows: P < 0.05 (*), P < 0.01 (**), P < 0.001 (***).

By RT-qPCR, five biomarkers, BCL2, CCR7, FAM162A, FOXO1, and RPS3, were notably downregulated in COPD and expression trends were consistent with GSE94916 and GSE42057 datasets (Figure 9).

## 4 Discussion

Chronic Obstructive Pulmonary Disease (COPD) is a condition characterized by chronic inflammation, leading to irreversible airway remodeling and disruption of airspace. This results in chronic bronchitis and emphysema. COPD is projected to become the third leading cause of death worldwide by 2030, yet effective treatments remain elusive (Mathers and Loncar, 2006; Racanelli and Choi, 2021). Smoking exposure is a major risk factor for COPD development, with subsequent imbalances in oxidative stress, inflammation, and growth factor signaling contributing to dysregulation and/or death of epithelial, endothelial, and immune cell compartments in the lung (Christenson et al., 2022). Programmed Cell Death (PCD) pathways are genetically encoded programs that maintain tissue homeostasis following cellular stress or injury. However, PCD can also represent an abnormal response in the pathogenesis of tissue damage, leading to harmful consequences in human diseases (Ashkenazi and Salvesen, 2014). Apoptosis, a typical form of PCD, involves caspase activation associated with chromatin condensation, cell shrinkage, DNA fragmentation, and eventual mitochondrial dysfunction. This study aims to identify potential and significant disease targets and biomarkers of COPD by investigating its classic pathogenesis and constructing a nomogram model based on these biomarkers for verification and further exploration of COPD pathogenesis.

In this study, five molecules—BCL2, CCR7, FAM162A, FOXO1, and RPS3—associated with the development of COPD were found to be involved in apoptosis and mitochondrial function. Their expression levels were significantly lower in epithelial sequencing data from COPD patients compared to the control group, demonstrating consistency. The B-cell lymphoma-2 gene (BCL2), the first member of the BCL2 family and a key target in apoptosis research, plays a dominant role in endogenous apoptosis and is essential for maintaining the balance between cell survival and cell death (Liu P. et al., 2023). Previous studies have clarified that Deregulation of apoptosis mediators’ p53 and bcl2 in lung tissue of COPD patients (Siganaki et al., 2010). RPS3 encodes a ribosomal protein belonging to the S3P family, which has an extraribosomal role as an endonuclease involved in repairing UV-induced DNA damage (Wan and Lenardo, 2010). Recent research has revealed that this gene interacts with the NF-κB p65 subunit, enhancing its DNA-binding activity and thereby contributing to the inflammation observed in COPD (Dong et al., 2018). FAM162A, located in the cytosol and mitochondria, is involved in several processes, including the activation of cysteine-type endopeptidase activity, which plays a role in cell apoptosis, response to hypoxia, and the release of cytochrome c from mitochondria (Schuhmann et al., 2024). FOXO1, or forkhead box O1, is a transcription factor expressed in various cell types. FOXO1 regulates cell proliferation, apoptosis, metabolism, stress, and other cellular activities by responding to internal and external signals, such as the activation of the PI3K/AKT signaling pathway (Xin et al., 2018). The protein encoded by the CCR7 gene activates CC chemokine receptors and participates in multiple processes, including the positive regulation of immune responses and leukocyte chemotaxis (Salem et al., 2021). Studies have shown that the expression level of CCR7 is downregulated during cuproptosis, suggesting that cuproptosis may be an important factor in altering CCR7 expression (Korbecki et al., 2020). Moreover, some articles have shown that CCR7 is low in COPD and can be used as a prognostic factor (Pan et al., 2023). PRD has long been implicated in lung remodeling and tissue destruction in COPD, particularly in emphysema. All the molecules mentioned inhibit PRD and mitochondrial dysfunction and are significantly underexpressed in COPD pathogenesis, indicating a disorder in the orderly PRD of epithelial cells.

The GSEA analysis of five biomarkers revealed that the co-enriched signaling pathways are “Ribosome,” “Lysosome,” “Endocytosis,” and “Epithelial Cell Signaling In.” These pathways all demonstrate processes closely related to the development of COPD. The “Ribosome” pathway has been associated with programmed cell death in several recent studies. Research indicates that ribosome stasis can trigger PRD (Tong et al., 2022). In response to DNA damage, specific genes inhibit the ribosome’s protein synthesis function, leading to ribosome stasis. This stagnation further initiates an apoptosis mechanism independent of the tumor protein p53 (Boon et al., 2024). The “Lysosome” signaling pathway has also been implicated in PRD across multiple studies. Apoptosis signaling pathways can be categorized into exogenous pathways (death receptor pathway) and endogenous pathways (mitochondrial pathway) (Tong et al., 2022). Both pathways converge at the downstream effector Caspase, which directly causes the degradation of vital proteins and activates nuclease during the execution phase of apoptosis, ultimately leading to cell death. Notably, the lysosomal pathway can induce apoptosis independently of the Caspase pathway under certain conditions.

The aberrant immune microenvironment in COPD patients is a significant etiological factor. Dysfunction of the adaptive immune system serves as a critical physiological basis for recurrent infections throughout the disease progression. Smoking and exposure to respiratory pathogens result in inflammatory cell infiltration, characterized by an increase in neutrophils and macrophages but a decrease in lymphocytes. Additionally, B cells exhibit impaired immunoglobulin secretion, leading to compromised adaptive immune function (Kheradmand et al., 2023; Hogg and Timens, 2009; Polverino, 2022). Our analysis revealed that the expression of BCL2, CCR7, FOXO1, and RPS3 is positively correlated with B-cell infiltration and negatively correlated with the M0 phase of macrophages and neutrophils. This finding suggests a relationship between these genes and the immune microenvironment of COPD, though the underlying mechanisms require further investigation.

Circular RNAs (circRNAs) are a class of non-coding RNAs that are widely present in eukaryotes and exhibit high stability and conservation due to their unique covalently closed circular structures (Duan et al., 2020). Previous studies have demonstrated that circRNAs play roles in the onset and progression of COPD through various mechanisms. Zhang Jun et al. employed gene ontology enrichment analysis, revealing that circRNAs may influence COPD development primarily by affecting cellular processes and molecular binding (Demedts et al., 2006). Additionally, circRNAs may regulate pathways such as non-homologous end joining, iron death, and FoxO signaling, thereby contributing to COPD pathogenesis (Kosmider et al., 2019; Yoshida et al., 2019). Duan et al. constructed networks including the circRNA target pathway, circRNA-miRNA-mRNA (ceRNA network), and functional ceRNA regulatory modules using microarray analysis, real-time quantitative PCR, and functional assays. They identified hsa-circRNA-0008672 as involved in NOD-like receptor signaling pathways, natural killer cell-mediated cytotoxicity, and Th17 cell differentiation, suggesting that circRNAs might impact the immune balance and contribute to COPD development (Duan et al., 2020). As a possible downstream of the biomarkers we identified, further investigation may be needed.

Our study identified BCL2, CCR7, FAM162A, FOXO1, and RPS3 as potential biomarkers for COPD, offering new insights into the treatment of this condition. However, at present, the verification of these findings in the external queue is insufficient. Only microarray-based sequencing data has been used, and the verification process needs to be further expanded. We have also comprehensively elucidated the mechanisms underlying these biomarkers and their roles in COPD formation, clarifying the pathogenesis of COPD through its enrichment pathways, immune microenvironment, circular RNA interactions, and other relevant aspects. This comprehensive understanding aims to improve clinical detection and treatment strategies for COPD.

## 5 Conclusion

In summary, BCL2, CCR7, FAM162A, FOXO1, and RPS3 are biomarkers for COPD, providing a new breakthrough point for the treatment of this disease.

## Data Availability

The datasets presented in this study can be found in online repositories. The names of the repository/repositories and accession number(s) can be found in the article/Supplementary Material.
