# Supplementary material for: Identification of biomarkers associated with mitochondrial dysfunction and programmed cell death in chronic obstructive pulmonary disease via transcriptomics
Source: Front Genet. 2025 Jun 19;16:1567173. doi: 10.3389/fgene.2025.1567173 (PMC12222144; doi:10.3389/fgene.2025.1567173)
Supplement: Supplementary file 1 [file Table1.DOCX]

| **Table S1 The primers of bimarkers for RT-qPCR** | |
| --- | --- |
| **genes** | **primers** |
| BCL21 F | CTTTGAGTTCGGTGGGGTCA |
| BCL21 R | AGCCCAGACTCACATCACCA |
| CCR7 F | GTTGGGCGTAAACGTGGACT |
| CCR7 R | TAGTCCTGCTGCGATTGGAC |
| FAM162A F | GTGACATTGAGCTCACCAGC |
| FAM162A R | CCGGGACTTTCCTGTGGTTT |
| FOXO1 F | AGGCTGGTTTTTCCATGTTGAC |
| FOXO1 R | TTTACACACAGGGCAAGCCA |
| RPS3 F | ACCACGTGAGCATTGTGGAA |
| RPS3 R | TCGCGTCTGTTCCCAAACAC |
| R-GAPDH F | GACCCCTTCATTGACCTCAAC |
| R-GAPDH R | GCCATCACGCCACAGCTTTCC |
