# Supplementary figures and images for: Identification of biomarkers associated with mitochondrial dysfunction and programmed cell death in chronic obstructive pulmonary disease via transcriptomics
Source: Front Genet. 2025 Jun 19;16:1567173. doi: 10.3389/fgene.2025.1567173 (PMC12222144; doi:10.3389/fgene.2025.1567173)

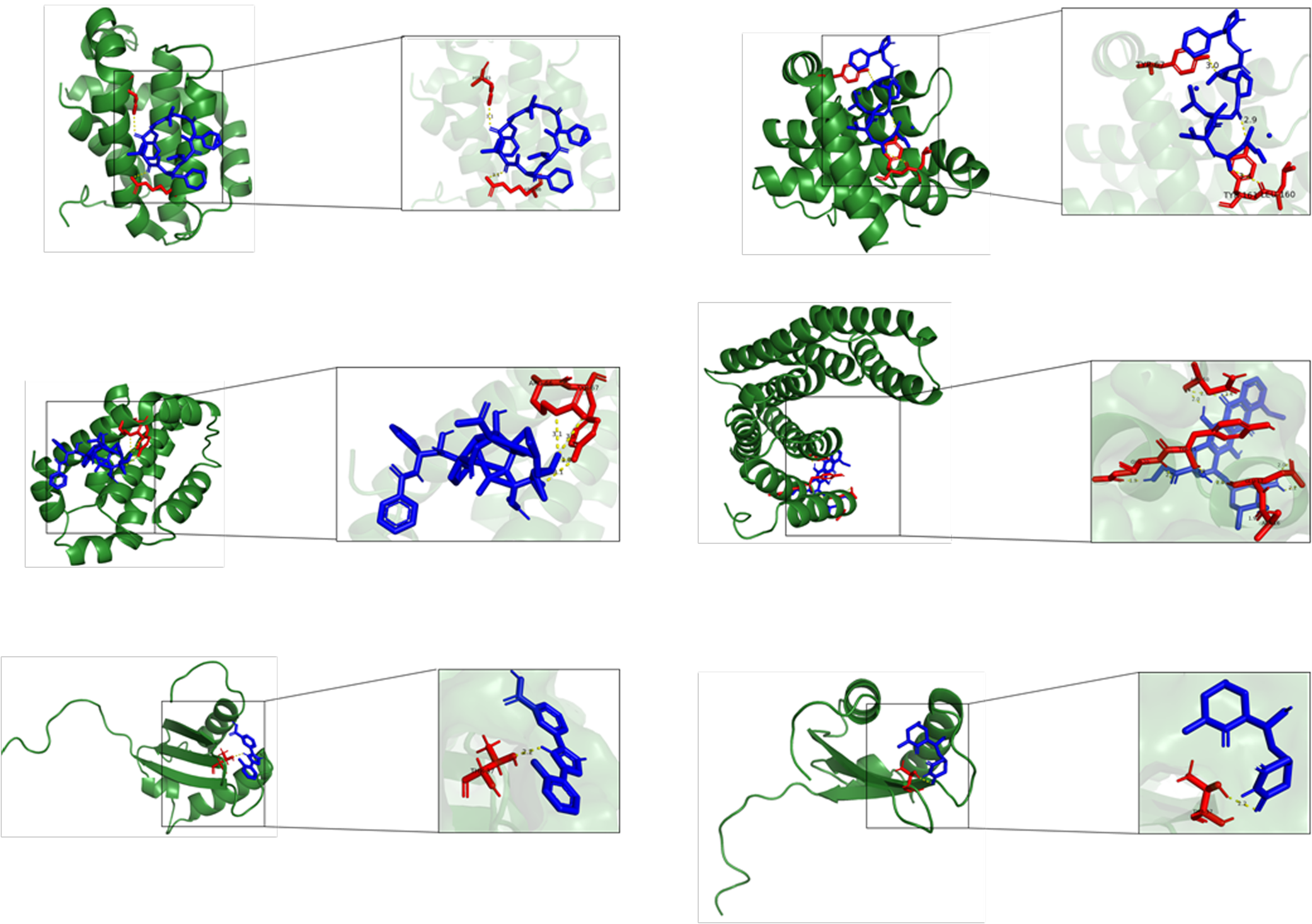

Supplement: Supplementary file 2 [file Image1.TIF]
